# Systemic Sclerosis Serum Steers the Differentiation of Adipose-Derived Stem Cells Toward Profibrotic Myofibroblasts: Pathophysiologic Implications

**DOI:** 10.3390/jcm8081256

**Published:** 2019-08-19

**Authors:** Mirko Manetti, Eloisa Romano, Irene Rosa, Bianca Saveria Fioretto, Emanuela Praino, Serena Guiducci, Florenzo Iannone, Lidia Ibba-Manneschi, Marco Matucci-Cerinic

**Affiliations:** 1 Section of Anatomy and Histology, Department of Experimental and Clinical Medicine, University of Florence, 50134 Florence, Italy; 2 Division of Rheumatology and Scleroderma Unit, AOUC, Department of Geriatric Medicine, Department of Experimental and Clinical Medicine, University of Florence, 50134 Florence, Italy; 3 Rheumatology Unit, Department of Emergency and Organ Transplantation, University of Bari, 70121 Bari, Italy

**Keywords:** systemic sclerosis, scleroderma, fibrosis, adipose-derived stem cells, myofibroblasts, myofibroblastic differentiation

## Abstract

Systemic sclerosis (SSc; scleroderma) is characterized by life-threatening progressive multiorgan fibrosis orchestrated by profibrotic myofibroblasts originating from different sources. Because recent data demonstrated that the majority of myofibroblasts in a murine scleroderma model arise from adipocytic progenitors through the adipocyte-myofibroblast transition process, we sought to determine whether the SSc microenvironment may affect the differentiation potential of adipose-derived stem cells (ADSC). Normal human ADSC from three donors were treated with serum from SSc patients (*n* = 6), serum from healthy individuals (*n* = 6), or recombinant human transforming growth factor-β1 (TGFβ1) as positive control of myofibroblastic phenotype induction. ADSC were subjected to in vitro adipogenic differentiation for up to 21 days in the presence of different stimuli followed by lipid content quantification. In selected experiments, adipocytic and mesenchymal/myofibroblast marker gene and protein expression levels were assessed by Real-Time PCR, immunoblotting and immunofluorescence after administration of different stimuli for 72 and 96 h, respectively. Cell contractile phenotype was assayed by collagen gel contraction assay. Likewise stimulation with TGFβ1, SSc serum was able to significantly inhibit the adipocyte differentiation of ADSC as testified by a strong decrease in red-colored lipid droplets after 21 days of adipogenic induction. Treatment of ADSC either with SSc serum or TGFβ1 resulted in the acquisition of a myofibroblast-like phenotype characterized by a reduced expression of the adipocytic markers perilipin and adiponectin, a significant upregulation of the mesenchymal/myofibroblast markers α-SMA+ stress fibers, S100A4 and type I collagen, and an ability to effectively contract collagen gels. In SSc, the pathologic environment may favor the differentiation of ADSC into profibrotic and contractile myofibroblast-like cells. These findings strengthen the notion that the generation of myofibroblasts from ADSC may be relevant in SSc pathophysiology potentially representing a new target for the prevention/treatment of multiorgan fibrosis.

## 1. Introduction

Systemic sclerosis (SSc; scleroderma) is a connective tissue disease characterized by dysregulation of innate and adaptive immunity, severe alterations in the microvasculature and progressive development of fibrotic lesions in the skin and internal organs [1,2,3]. The key cellular players responsible for SSc-related fibrosis are activated myofibroblasts, a population of mesenchymal cells that exhibit both the extracellular matrix (ECM)-synthesizing features of fibroblasts and the cytoskeletal characteristics of contractile smooth muscle cells [4,5,6]. Profibrotic myofibroblasts display an increased production of fibrillar type I and type III collagens, as well as the expression and incorporation into stress fibers of α-smooth muscle actin (α-SMA) and a shortage of ECM-degrading enzymes [4,5,6]. In the skin of SSc patients, the presence of myofibroblasts correlates with a number of parameters of fibrosis such as tightness, hardness and stiffness, supporting a role for these profibrotic cells in different disease clinical signs [4]. Besides the skin, in SSc myofibroblasts can be observed in any organ affected by fibrosis including the lungs, gastrointestinal tract, kidneys, and myocardium [7,8,9,10].

Although tissue accumulation of myofibroblasts and the persistence of their elevated biosynthetic functions are regarded as crucial determinants of the extent and rate of progression of the SSc-related fibrotic process, the initial pathogenic events leading to the appearance of these cells remain a matter of debate [1,4,5]. Indeed, increasing evidence suggests that myofibroblasts may arise from different sources including expansion and activation of resident tissue fibroblasts and perivascular pericytes, recruitment of bone marrow-derived circulating precursors, and transdifferentiation of epithelial and endothelial cells [6,11,12,13]. Interestingly, recent studies have also revealed that, in the course of fibrosis, adipocyte progenitors (i.e., preadipocytes) may transdifferentiate into contractile myofibroblasts through a process termed adipocyte-myofibroblast transition [14,15,16]. In fact, by using a transgenic lineage tracing approach in experimental murine scleroderma, it was possible to demonstrate that the loss of intradermal adipose tissue precedes the onset of dermal fibrosis and is accompanied by early downregulation of adipocyte-associated genes followed by an acquisition of myofibroblast-associated genes [14]. Strikingly, development of skin fibrosis in SSc patients is accompanied by loss of dermal white adipose tissue [15], a phenotype well recapitulated in different animal models of skin fibrosis [17,18,19,20,21,22,23]. Moreover, adipose tissue replacement with fibrotic tissue has been observed in other pathologies such as liver fibrosis and cancers [24,25,26,27,28]. Of note, it has been reported that stimulation of either undifferentiated human adipose-derived stem cells (ADSC) or ADSC committed to preadipocytes with profibrotic transforming growth factor-β (TGFβ) is able to induce the loss of adipocytic markers and the reciprocal acquisition of mesenchymal/myofibroblast markers [14,29]. In vitro experiments on different adipocyte-generating cultures further showed that myofibroblast differentiation may be driven not only by TGFβ [14], but also by other pathways such as FIZZ1 and Wnt signaling [23,30,31].

Several studies have also suggested that SSc patients may benefit from fat tissue grafting thanks to its biocompatibility, property of filling and high content of multipotent mesenchymal-like stem/progenitor cells, such as ADSC, that reside in the so-called stromal vascular fraction (SVF) and may display regenerative, anti-inflammatory, immunosuppressive, and proangiogenic activities [32,33,34,35,36,37]. In a model of scleroderma-like skin sclerosis in nude mice, subcutaneous injections of SVF significantly decreased dermal fibrosis and increased local vascularization [38]. Autologous fat implantation in hands of patients with SSc led to significant pain relief and decrease in the number, duration, and severity of Raynaud’s phenomenon attacks [39]. An improvement of digital ulcers was also reported in a small group of SSc patients after autologous adipose-derived SVF injection [40]. Furthermore, a significant reduction in skin tightening was observed in SSc patients subjected to transplantation of ADSC in hyaluronic acid solution [41]. Encouraging results on the safety, tolerability and potential efficacy of autologous adipose-derived SVF injection to treat hand disability in SSc patients have also emerged from a small clinical trial and subsequent follow-up analyses [42,43,44]. Although the aforementioned reports suggest that autologous SVF might be a valuable source of therapeutic stem/progenitor cells, the exact role of the ADSC contained within the SVF could not be established leaving undetermined whether these cells may be optimally effective in SSc patients. Indeed, a recent study reported that SSc SVF-derived ADSC, though retaining high multipotency, fail to differentiate into fully mature adipocytes, osteocytes and chondrocytes [45]. A differential molecular signature reflecting deregulation of angiogenesis, endothelial activation and fibrosis was also detected in SSc SVF when compared with healthy SVF [46]. As previously reported for SSc bone marrow-derived mesenchymal stem/stromal cells [47], in vitro-expanded SSc ADSC at early passages have been found to display a profibrotic and anti-adipogenic phenotype [29]. Collectively, these data suggest that the specific pathologic tissue environment may be crucial in affecting the differentiation potential and behavior of ADSC.

On this background, the objective of our in vitro study was to investigate for the first time how and in which extent the SSc microenvironment may influence the fate of human ADSC. In particular, we aimed at comprehensively exploring whether treatment with sera from SSc patients may either affect the differentiation of normal human ADSC into mature adipocytes or promote their differentiation toward profibrotic myofibroblasts.

## 2. Materials and Methods

### 2.1. Patients and Serum Samples

Serum samples were obtained from 6 patients with early diffuse cutaneous SSc (dcSSc; disease duration <2 years from first non-Raynaud symptom) fulfilling the American College of Rheumatology/European League Against Rheumatism 2013 classification criteria [48] and from 6 age- and sex-matched healthy volunteers. SSc patients were not on immunosuppressors, corticosteroids or other disease-modifying drugs. Before blood sampling, patients were washed out for 10 days from oral vasodilating drugs and for 2 months from intravenous prostanoids. Fresh venous blood samples were drawn, left to clot for 30 min before centrifugation at 1500× *g* for 15 min, and serum was collected and stored in aliquots at −80°C until used. The study was conducted according to the principles of the Declaration of Helsinki and approved by the local institutional review board at the Azienda Ospedaliero-Universitaria Careggi (AOUC), Florence, Italy (AOUC 69/13). All individuals provided written informed consent.

### 2.2. Culture of Human ADSC

Three lines of normal human ADSC were purchased from Lonza (catalog no. PT-5006; Lonza, Basel, Switzerland) and were routinely maintained in ADSC complete medium (ADSC human Adipose Derived Stem Cell Growth BulletKit^TM^ Medium; catalog no. PT-4505; Lonza) at 37 °C in a 5% CO_2_ incubator.

### 2.3. In Vitro Differentiation of Human ADSC into Mature Adipocytes

ADSC were seeded into 6-well culture plates at a density of 2 × 10^5^ cells/well in ADSC complete medium and cultured for 6 days. Once at confluence, cells were treated with fresh adipogenic induction medium (ADSC complete medium supplemented with 1 μM dexamethasone, 0.5 mM 3-isobutyl-1-methylxanthine, 60 μM indomethacin, and 10 μg/mL recombinant human insulin) for 72 h and, subsequently, with fresh adipogenic maintenance medium (ADSC complete medium with 10 μg/mL recombinant human insulin) for 24 h in the presence of the following stimuli: recombinant human TGFβ1 (10 ng/mL; PeproTech, Rocky Hill, NJ, USA) or 10% serum from patients with early dcSSc (*n* = 6) and healthy individuals (*n* = 6). Each serum sample was tested individually. This protocol was repeated up to 3 weeks. ADSC were characterized by Oil red O staining at 7, 14, and 21 days following adipogenic induction medium/maintenance medium plus stimuli addition.

### 2.4. Oil Red O Staining

Oil red O powder was obtained from Sigma-Aldrich (catalog no. O0625; Sigma-Aldrich, St. Louis, MO, USA). A stock solution was prepared by dissolving 50 mg of Oil red O powder in 50 mL 2-propanol and diluted with distilled water (2:3 ratio) to obtain a working solution. ADSC were fixed with 4% formaldehyde in phosphate buffered saline (PBS) for 20 min at room temperature. Formaldehyde was removed and Oil red O working solution was added to the 6-well plates for 10 min at room temperature. After washing with distilled water phase-contrast images were captured using a Leica inverted microscope (Leica Microsystems, Mannheim, Germany) equipped with a digital camera. Oil red O staining was quantified after 21 days by adding 100% 2-propanol (2.5 mL per well) to elute the dye followed by plate incubation for 10 min at room temperature on an orbital shaker. For each experimental point, 400 μL of eluate were transferred into two wells of a 96-well microtiter plate (200 μL per well). Absorption was measured in duplicate at 510 nm in a Promega GloMax Multi Detection Microplate Reader (Promega Corporation, Madison, WI, USA).

### 2.5. Stimulation of Human ADSC to Assess Myofibroblastic Differentiation

In selected experiments, ADSC were grown to 70% confluence, washed three times with serum-free medium and cultured in ADSC basal medium (catalog no. PT-3273, Lonza) supplemented with 2% fetal bovine serum for 24 h. Medium was removed and cells were then incubated in ADSC basal medium supplemented with 2% fetal bovine serum and recombinant human TGFβ1 (10 ng/mL; PeproTech) or 10% serum from patients with early dcSSc (*n* = 6) and healthy individuals (*n* = 6) for 72 and 96 h for subsequent analyses of mRNA and protein expression levels of relevant markers, respectively. Each serum sample was tested individually. The medium was changed, and additives replenished, every day.

### 2.6. RNA Isolation and Quantitative Real-Time PCR

Seventy-two h after stimulation, ADSC were harvested and total RNA was isolated using the RNeasy Micro Kit (Qiagen, Milan, Italy). First strand cDNA was synthesized using the QuantiTect Reverse Transcription kit (Qiagen). For mRNA quantification, SYBR Green Real-Time PCR was performed using the StepOnePlus Real-Time PCR System (Applied Biosystems, Milan, Italy) with melting curve analysis. Predesigned oligonucleotide primer pairs were obtained from Qiagen (QuantiTect Primer Assay). The assay IDs were: Hs_PLIN1_1_SG (perilipin-1; catalog no. QT00017486), Hs_ADIPOQ_va.1_SG (adiponectin; catalog no. QT02409862), Hs_ACTA2_1_SG (α-SMA; catalog no. QT00088102), Hs_S100A4_1_SG (S100 calcium binding protein A4; catalog no. QT00014259), Hs_COL1A1_1_SG (collagen, type I, alpha 1; catalog no. QT00037793), Hs_COL1A2_1_SG (collagen, type I, alpha 2; catalog no. QT00072058), and Hs_RRN18S_1_SG (18S ribosomal RNA; catalog no. QT00199367). 18S ribosomal RNA was measured as an endogenous control to normalize for the amounts of loaded cDNA. Differences were calculated with the threshold cycle (Ct) and comparative Ct method for relative quantification. All measurements were performed in triplicate.

### 2.7. Fluorescence Immunocytochemistry

ADSC were seeded onto glass coverslips and stimulated as already described for 96 h. Cells were then fixed with 3.7% buffered paraformaldehyde and permeabilized with 0.1% Triton X-100 in PBS. Slides were washed with PBS and blocked with 1% bovine serum albumin in PBS for 1 h at room temperature. Double immunofluorescence staining was performed overnight at 4 °C by mixing mouse and rabbit primary antibodies. The following primary antibodies were used: Rabbit polyclonal anti-perilipin-1 (1:80 dilution; catalog no. ab3526; Abcam, Cambridge, UK), mouse monoclonal anti-α-SMA (1:50 dilution; catalog no. ab7817; Abcam), mouse monoclonal anti-type I collagen (1:100 dilution; catalog no. ab90395; Abcam), mouse monoclonal anti-adiponectin (1:100 dilution; catalog no. ab22554; Abcam) and rabbit monoclonal anti-S100A4/fibroblast-specific protein-1 (1:100 dilution; catalog no. ab124805; Abcam). Irrelevant isotype-matched and concentration-matched mouse and rabbit IgG (Sigma-Aldrich) were used as negative controls. The day after, cells were incubated for 45 min at room temperature in the dark with Alexa Fluor-488-conjugated or Rhodamine Red-X-conjugated secondary antibodies at 1:200 dilution (Invitrogen, Carlsbad, CA, USA). Nuclei were counterstained with 4′, 6-diamidino-2-phenylindole (DAPI). Immunostained cells were examined with a Leica DM4000 B microscope (Leica Microsystems) and fluorescence images were captured with a Leica DFC310 FX 1.4-megapixel digital color camera equipped with the Leica software application suite LAS V3.8 (Leica Microsystems).

### 2.8. Immunoblotting

Whole cell protein lysates from ADSC were collected 96 h after stimulation and subjected to immunoblot analysis as described elsewhere [6]. The following primary antibodies from Abcam were used: anti-perilipin-1 (1:500 dilution; catalog no. ab3526), anti-adiponectin (1:1000 dilution; catalog no. ab22554), anti-α-SMA (1:500 dilution; catalog no. ab7817), anti-type I collagen (1:500 dilution; catalog no. ab90395), and anti-S100A4 (1:1000 dilution; catalog no. ab124805). Anti-α-tubulin antibody (1:1000 dilution; catalog no. #2144; Cell Signaling Technology, Danvers, MA, USA) was used for normalization. Immunodetection was performed using the Western Breeze Chromogenic Western Blot Immunodetection Kit (Invitrogen). Band intensities were quantified with the free-share ImageJ software 64-bit Java 1.8.0_112 Windows version (NIH, Bethesda, MD, USA; online at http://rsbweb.nih.gov/ij) and normalized with the respective α-tubulin values.

### 2.9. Collagen Gel Contraction Assay

Collagen gel contraction assay was performed using the CytoSelect^TM^ 24-Well Cell Contraction Assay Kit (Floating Matrix Model; catalog no. CBA-5020; Cell Biolabs, San Diego, CA, USA) according to the manufacturer’s instructions. ADSC were cultured in basal medium supplemented with 2% fetal bovine serum for 24 h and then stimulated with recombinant human TGFβ1 (10 ng/mL; PeproTech) or 10% serum from early dcSSc patients (*n* = 6) and healthy individuals (*n* = 6) for 96 h before the assay. After treatment, ADSC were harvested, pelleted and resuspended in serum-free medium at 3 × 10^6^ cells/mL. For each experimental point, 100 µL of the cell suspension was mixed with 400 µL of cold neutralized collagen gel solution and subsequently added to one well of the adhesion resistant matrix-coated 24-well cell contraction plate. Gels were allowed to solidify for 1 h at 37°C and 5% CO_2_. After polymerization, 1 mL of basal medium or medium containing different stimuli (i.e., recombinant human TGFβ1, early dcSSc sera and healthy sera) was added to the top of each collagen gel lattice. Gels without cells were included as negative controls. Each experimental point was performed in triplicate. After 24 h, the culture dish was scanned and the area of each collagen gel was measured by ImageJ software 64-bit Java 1.8.0_112 Windows version (NIH).

### 2.10. Determination of TGFβ1 Serum Levels

Levels of TGFβ1 in serum samples were measured by commercial quantitative colorimetric sandwich enzyme-linked immunosorbent assay (ELISA) according to the manufacturer’s recommendation (eBioscience, San Diego, CA, USA). The detection limit of the assay according to the manufacturer was 8 pg/mL and the standard curve covered a concentration range from 8 to 1000 pg/mL. Concentrations were calculated using a standard curve generated with specific standards provided by the manufacturer. Each sample was tested in triplicate.

### 2.11. Statistical Analysis

Statistical analyses were performed using the Statistical Package for Social Sciences software for Windows, V.25.0 (SPSS, Chicago, IL, USA). Data are reported as the mean ± SD or SEM. Student’s *t*-test was used for statistical evaluation of the differences between two independent groups. Values of *p* < 0.05 according to a two-tailed distribution were considered statistically significant.

## 3. Results

### 3.1. Treatment with SSc Serum Significantly Inhibits the Adipogenic Differentiation Process of Normal Human ADSC

**Figure 1:**
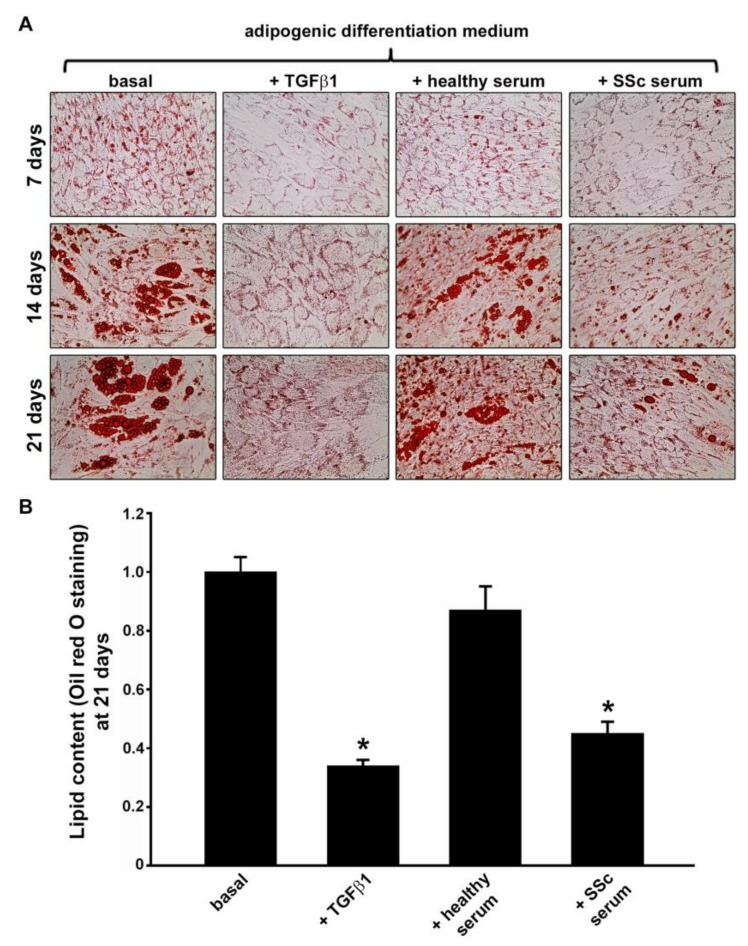
Treatment with serum from patients with systemic sclerosis (SSc) significantly reduces the lipid content in normal human adipose-derived stem cells (ADSC) cultured in adipogenic differentiation medium. (**A**) Confluent ADSC were treated with fresh adipogenic induction medium for 72 h and, subsequently, with fresh adipogenic maintenance medium together with different stimuli (i.e., recombinant human transforming growth factor-β1 (TGFβ1; 10 ng/mL), 10% serum from healthy individuals (*n* = 6) or 10% serum from patients with early diffuse cutaneous SSc (*n* = 6)) for 24 h. This protocol was repeated up to 3 weeks. Cells were subjected to Oil red O staining at 7, 14 and 21 days following adipogenic induction medium/maintenance medium plus stimuli addition. Representative images of Oil red O staining for each experimental point are shown. (**B**) The relative lipid content values at 21 days are expressed as mean ± SEM of three independent experiments performed with three ADSC lines. Basal ADSC lipid content was set to 1; the other results are expressed as x-fold change over basal ADSC lipid content. Student’s *t*-test was used for statistical analysis. * *p* < 0.001 vs. basal and healthy serum.

Oil red O staining was employed to monitor the accumulation of intracellular lipids at 7, 14, and 21 days of ADSC culture using a protocol directing differentiation into the mature adipocyte phenotype in the presence of different stimuli. At all three experimental time points, under the microscope, normal human ADSC from three donors challenged with recombinant human TGFβ1 and serum from patients with SSc showed decreased red-colored lipid droplets compared to both basal and healthy serum-treated ADSC (Figure 1A). Quantitative analysis of intracellular lipid accumulation revealed that after 21 days of culture in adipogenic differentiation medium in the presence of SSc sera, the lipid content was strongly reduced compared with either cells in basal conditions or cells cultured in the presence of healthy sera (*p* < 0.001 for both comparisons) (Figure 1B). Moreover, the lipid content of cells treated with SSc sera was comparable to that of cells challenged with recombinant human TGFβ1 (Figure 1B). Levels of TGFβ1 measured by ELISA were significantly higher in SSc serum samples (mean ± SD values, 86.55 ± 23.12 pg/mL) than in healthy control serum samples (mean ± SD values, 31.24 ± 17.64 pg/mL; *p* < 0.001).

### 3.2. Treatment with SSc Serum Induces a Myofibroblast-Like Profibrotic and Contractile Phenotype in Normal Human ADSC

Previous studies suggested that ADSC and adipocyte progenitors/preadipocytes can potentially contribute to myofibroblast accumulation in the fibrotic skin of SSc patients [14,15,16,29]. Nevertheless, whether the SSc microenvironment may influence the fate of normal human ADSC has never been investigated. To address this issue, normal human ADSC were challenged with sera from patients with SSc and healthy individuals and subsequently assayed for changes in gene and protein expression of adipocytic and mesenchymal/myofibroblast markers. According to the literature [14,29], stimulation with recombinant human TGFβ1 was performed in parallel as a positive control of myofibroblastic phenotype induction.

**Figure 2:**
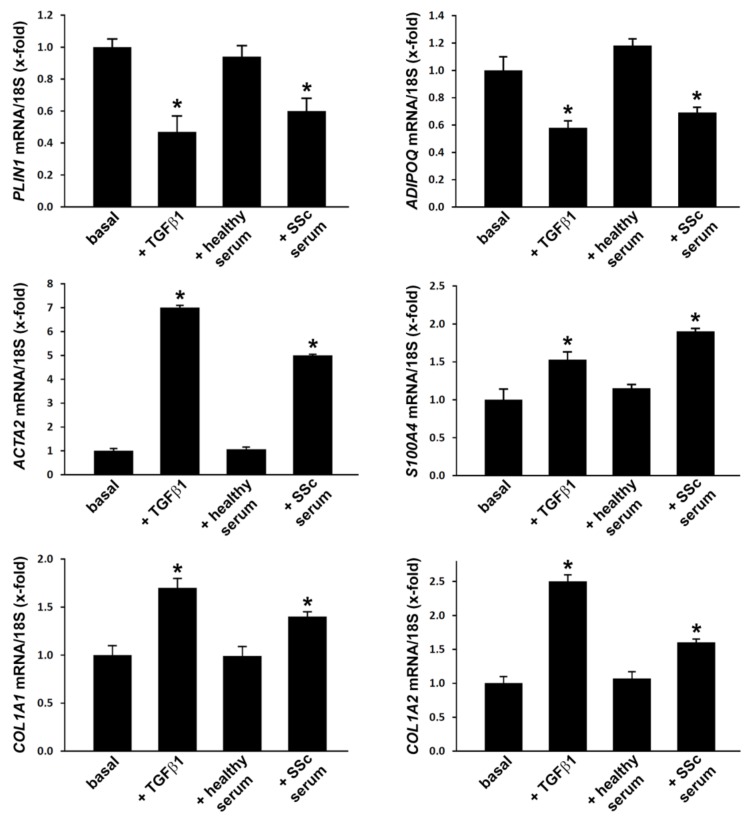
Treatment with systemic sclerosis (SSc) serum induces changes in gene expression levels of adipocytic and mesenchymal/myofibroblast markers in normal human adipose-derived stem cells (ADSC). ADSC were treated for 72 h with recombinant human transforming growth factor-β1 (TGFβ1; 10 ng/mL), 10% serum from healthy individuals (*n* = 6) or 10% serum from patients with early diffuse cutaneous SSc (*n* = 6) and subsequently assayed for mRNA expression levels of *PLIN1* (perilipin), *ADIPOQ* (adiponectin), *ACTA2* (α-smooth muscle actin), *S100A4* (fibroblast-specific protein-1), *COL1A1* and *COL1A2* (α1 and α2 chains of type I collagen, respectively) by quantitative Real-Time PCR. 18S ribosomal RNA was measured as an endogenous control for normalization. For each gene, the mRNA expression in basal cells was set to 1; the other results are expressed as x-fold increase/decrease over basal response. Bars represent the mean ± SEM. Results are representative of three independent experiments performed with three ADSC lines. Statistical analysis was carried out with Student’s *t*-test. * *p* < 0.001 vs. basal and healthy serum.

After 72-h stimulation of ADSC with SSc sera, the gene expression levels of the adipocytic markers *PLIN1* (perilipin-1) and *ADIPOQ* (adiponectin) assessed by quantitative Real-Time PCR were significantly decreased compared with either cells in basal conditions or cells treated with healthy sera (*p* < 0.001 for both comparisons) (Figure 2). In parallel, we detected a significant upregulation of genes encoding mesenchymal/myofibroblast markers, including *ACTA2* (α-SMA), *S100A4* (fibroblast-specific protein-1), *COL1A1* and *COL1A2* (α1 and α2 chains of type I collagen) in ADSC challenged with SSc sera (*p* < 0.001 vs. basal and healthy serum-treated ADSC for all genes) (Figure 2). As expected, treatment of ADSC with recombinant human TGFβ1 significantly downregulated both *PLIN1* and *ADIPOQ* and upregulated *ACTA2*, *S100A4*, *COL1A1*, and *COL1A2* gene expression levels (Figure 2).

**Figure 3:**
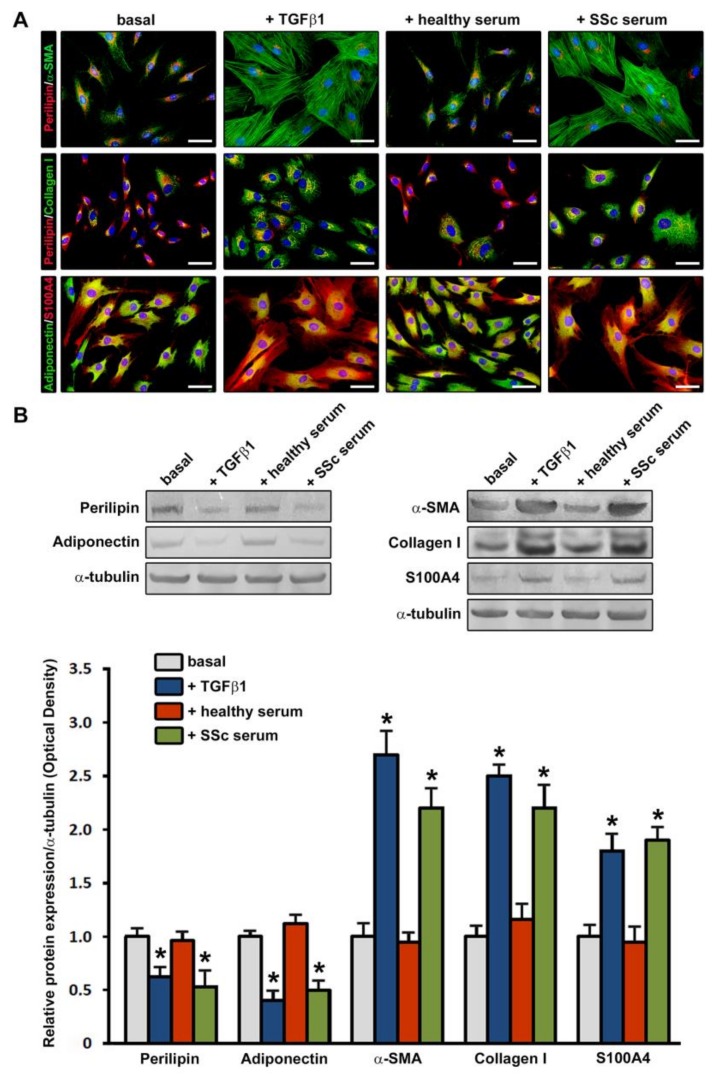
Treatment with serum from patients with systemic sclerosis (SSc) induces changes in protein expression levels of adipocytic and mesenchymal/myofibroblast markers in normal human adipose-derived stem cells (ADSC). ADSC were treated for 96 h with recombinant human transforming growth factor-β1 (TGFβ1; 10 ng/mL), 10% serum from healthy individuals (*n* = 6) or 10% serum from patients with early diffuse cutaneous SSc (*n* = 6) and subsequently assayed for protein expression levels of adipocytic markers (perilipin and adiponectin) and mesenchymal/myofibroblast markers (α-smooth muscle actin (α-SMA), type I collagen and S100A4/fibroblast-specific protein-1). (**A**) Representative fluorescence microphotographs showing ADSC double immunostained for perilipin (red) and α-SMA (green), perilipin (red) and type I collagen (green), and adiponectin (green) and S100A4 (red) are shown in the upper, middle and lower panels, respectively. Nuclei are counterstained with 4′,6-diamidino-2-phenylindole (DAPI; blue). Treatment of ADSC either with SSc sera or TGFβ1, but not with healthy sera, induces downregulation of perilipin and adiponectin in parallel with an increase in α-SMA+ stress fibers, type I collagen and S100A4. Scale bar = 50 μm. (**B**) Representative immunoblots are shown. α-tubulin was measured as a loading control. Results are representative of three independent experiments performed with three ADSC lines. The densitometric analysis of the bands normalized to α-tubulin is reported in the histograms. Data are mean ± SEM of optical density. Student’s *t*-test was used for statistical analysis. * *p* < 0.001 vs. basal and healthy serum for each protein.

These differences were maintained at the protein level as assessed by immunofluorescence and immunoblotting analyses of adipocytic and mesenchymal/myofibroblast markers in cells stimulated with sera for 96 h (Figure 3A,B).

As displayed in Figure 3A, ADSC were confirmed to be immunopositive for perilipin and adiponectin, although the immunostaining for these adipocytic markers was markedly decreased in cells challenged with SSc sera respect to those at basal conditions or stimulated with healthy sera. Moreover, immunofluorescence staining revealed a strong increase in α-SMA+ stress fibers and immunopositivity for type I collagen and S100A4 mesenchymal/myofibroblast markers in SSc serum-treated ADSC compared with both basal and healthy serum-treated ADSC (Figure 3A). The immunophenotypic changes of cells stimulated with SSc sera were comparable to those of cells treated with recombinant human TGFβ1 (Figure 3A).

Immunoblotting analyses confirmed either significantly lower protein levels of perilipin and adiponectin or significantly higher protein levels of α-SMA, type I collagen and S100A4 in both ADSC challenged with SSc sera and those treated with recombinant human TGFβ1 compared with basal ADSC and ADSC treated with healthy sera (*p* < 0.001 for all markers) (Figure 3B).

**Figure 4:**
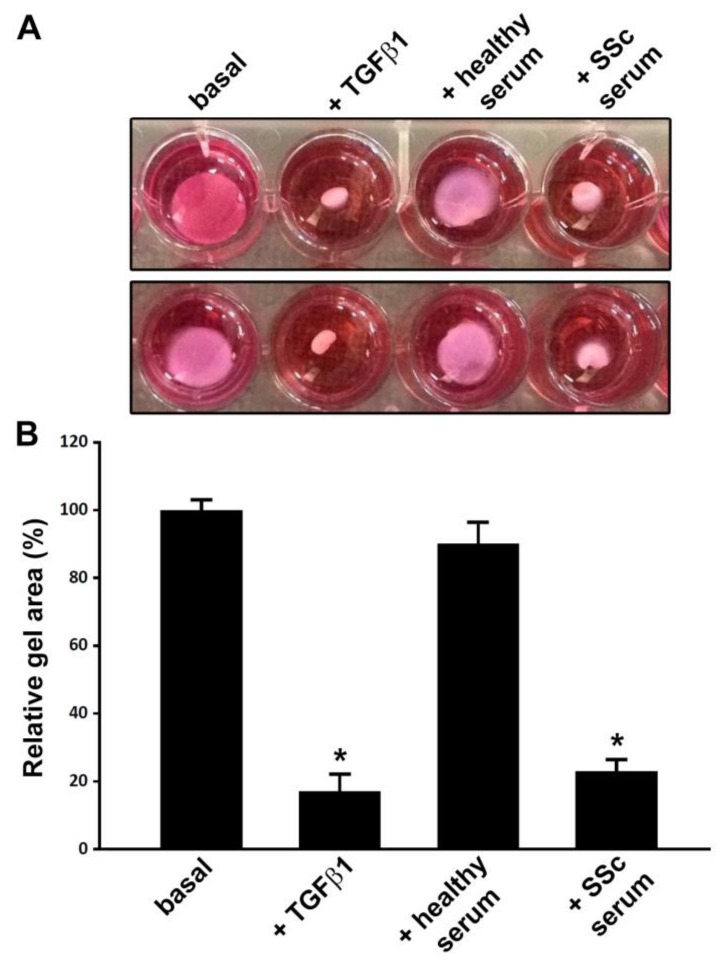
Normal human adipose-derived stem cells (ADSC) treated with serum from systemic sclerosis (SSc) patients acquire the ability to effectively contract collagen gel. (**A**) Representative images of collagen gel contraction assay with ADSC at basal conditions and after treatment with recombinant human transforming growth factor-β1 (TGFβ1; 10 ng/mL), 10% serum from healthy individuals (*n* = 6) or 10% serum from patients with early diffuse cutaneous SSc (*n* = 6) for 96 h. Results are representative of three independent experiments performed with three ADSC lines. (**B**) Gel area expressed as percentage of that observed in basal ADSC. Data are mean ± SEM. Statistical analysis was carried out with Student’s *t*-test. * *p* < 0.001 vs. basal and healthy serum.

The ability of SSc serum to induce a myofibroblastic differentiation of ADSC was confirmed functionally by the collagen gel contraction assay. In fact, similarly to stimulation with recombinant human TGFβ1, 96-h treatment with SSc sera was able to fully promote the acquisition of a myofibroblast-like contractile phenotype, as demonstrated by a significant reduction in collagen gel area (*p* < 0.001 vs. basal and healthy serum-treated ADSC) (Figure 4A,B).

## 4. Discussion

Our data demonstrate for the first time that normal human ADSC can acquire an anti-adipogenic and profibrotic phenotype in response to treatment with serum of SSc patients, suggesting that a preferential differentiation of these stem cells into myofibroblasts may take place in SSc and contribute to the reduction in dermal white adipose tissue and its replacement by fibrotic tissue. In fact, a prolonged challenge with SSc sera was able to significantly inhibit the adipocyte differentiation process of ADSC as testified by a strong decrease in red-colored lipid droplets after 21 days of adipogenic induction. Moreover, in parallel experiments SSc serum-treated ADSC not only exhibited a reduced expression of the adipocytic markers perilipin and adiponectin together with a significant upregulation of mesenchymal/myofibroblast markers, such as α-SMA+ stress fibers, S100A4 and type I collagen, but also functionally acquired a myofibroblast-like contractile phenotype. Since adiponectin has recently been shown to act as an endogenous anti-fibrotic mediator [49], we speculate that the decrease in adiponectin expression might have a prominent role in the SSc serum-induced acquisition of myofibroblastic features by ADSC. Of note, the results of the present study are in line with previous in vitro findings from our group demonstrating that sera from SSc patients may exert a profibrotic effect on another kind of cells, namely the transdifferentiation of dermal microvascular endothelial cells into myofibroblasts [6]. Furthermore, another report demonstrated that exosomes purified from the serum of SSc patients can effectively induce a profibrotic phenotype in cultured normal dermal fibroblasts [50]. Focusing on adipose tissue cells, both ADSC isolated from the abdominal fat of SSc patients and ADSC harvested from bleomycin-treated mice were found to display an anti-adipogenic/profibrotic phenotype, including a reduced capacity for adipogenic differentiation and an increased expression of the myofibroblast marker α-SMA respect to healthy ADSC [29]. In addition, it is worth noting that previous studies have clearly shown that a myofibroblastic phenotype is inducible in cultured undifferentiated ADSC or ADSC-derived preadipocytes by treatment with TGFβ, which is considered a leading profibrotic orchestrator in SSc pathogenesis through both canonical and non-canonical signaling pathways [1,14,29]. In this regard, the herein demonstration that changes in the phenotypic and functional characteristics of normal ADSC upon treatment with SSc serum and recombinant TGFβ are comparable further supports that the peculiar SSc pathologic environment may direct the differentiation of ADSC toward a profibrotic fate. Indeed, we verified that TGFβ1 levels were raised in SSc sera, though we believe that multiple serum factors may likely contribute to induce an anti-adipogenic and profibrotic phenotype of ADSC. Anyway the role of TGFβ may be dominant in this context, as also suggested by the recent evidence that this pleiotropic cytokine is even capable of promoting age-related adipogenic-to-fibrotic switch of dermal fibroblasts [51]. Consistent with the aforementioned in vitro observations, the notion that adipose tissue-resident stem/progenitor cells may contribute to the accumulation of fibrogenic myofibroblasts and the development of skin sclerosis is substantiated by cell fate mapping studies in the most widely used preclinical model of SSc, namely mice with bleomycin-induced scleroderma, where the majority of pathogenic myofibroblasts were found to arise from adiponectin-positive progenitors resident in the dermal white adipose tissue [14,15]. Interestingly, it appears that mature adipocytes may represent an additional source of profibrotic myofibroblasts [52]. In particular, it has recently been proposed that, through a two-step process, mature adipocytes de-differentiate into a kind of preadipocytes (referred to as adipose-derived preadipocytes) that then transdifferentiate into myofibroblasts [52]. Of note, this novel scenario is fully consistent with the evidence that in bleomycin-induced skin fibrosis the process of myofibroblast production and fibrosis is secondary to significant reduction of the dermal adipose tissue and, therefore, will deserve a thorough investigation in SSc.

In a context in which the adipose-derived SVF is receiving growing attention as an innovative biotherapy for SSc because of its abundance of stem/progenitor cells, of its accessibility and easy harvesting [42,43,44], our present results, together with those of previous works [14,15,29], highlight that an in-depth understanding of the differentiation potential and behavior of ADSC in a diseased milieu should not be overlooked. Indeed, the composition of adipose-derived SVF is very heterogeneous as, although ADSC represent its major constituents, it includes also endothelial cells/progenitors, pericytes, and hematopoietic and immune cells that may provide numerous mechanisms responsible for its therapeutic effects [46]. Specifically, several data published over the last few years support the possible clinical efficacy of regional implantation of autologous adipose-derived SVF to treat hand disability or promote healing of digital ulcers in SSc patients [40,41,42,43,44]. In a recent study that investigated the cellular and molecular profiles of therapeutic-grade adipose-derived SVF, the distribution of the various cell subpopulations was not different between patients with SSc (15 with limited cutaneous SSc and 9 with dcSSc) and healthy donors, and SSc SVF exhibited only slightly impaired vasculogenic/angiogenic potential either in vitro or in vivo [46]. However, a differential endothelial and stromal cell molecular signature of the adipose-derived SVF from SSc patients reflecting endothelial activation, deregulation of angiogenesis and fibrosis was revealed by global and single-cell RNA-sequencing and analyses of SVF-derived secreted factors [46]. In addition, another study analyzed the composition of adipose-derived SVF from 6 patients with dcSSc and found a reduced number of cells with a stem-like phenotype, a high content of proinflammatory cytokines and a shortage of angiogenic factors [45]. Furthermore, SSc SVF-derived ADSC were shown to retain high multipotency, but failed to sustain terminally differentiated adipocyte, osteocyte and chondrocyte progenies [45]. At variance with the aforementioned findings, other two studies comparing the properties of ADSC isolated from SSc patients with their healthy counterpart either reported similar proliferation, differentiation and immunosuppressive potentials [53], or revealed identical differentiation capacity but reduced proliferation and migration [54]. A very recent report has also shown that explanted SSc ADSC have profibrotic and anti-adipogenic features, including high levels of the myofibroblast marker α-SMA and low expression of both anti-fibrotic caveolin-1 and the adipogenic marker FABP4 [29]. Collectively, such discrepant findings derived from these relatively small studies might be related to differences in ADSC isolation, culture protocols and proliferative stage in culture, as well as the well-known clinical heterogeneity of SSc.

## 5. Conclusions

In summary, our data suggest that the SSc pathologic milieu may favor the differentiation of normal ADSC into profibrotic myofibroblast-like cells. This evidence may add up to recent data on ADSC isolated from SSc patients and murine skin fibrosis [29], strengthening the notion that adipose tissue-resident stem/progenitor cells may be relevant in SSc pathophysiology and might represent new targets for the prevention and treatment of multiorgan fibrosis. In perspective, further work is warranted to help in deciphering the specific systemic factors and signaling pathways that in SSc may drive the differentiation of ADSC into pathogenic myofibroblasts, potentially representing novel therapeutic targets. Our findings also encourage further investigation into the understanding of how ADSC can impact upon tissue fibrosis. Indeed, the possibility that ADSC populating the adipose-derived SVF may behave as a source of profibrotic myofibroblasts should be carefully considered when planning SVF-based therapeutic approaches in SSc patients. Therefore, only an in-depth cellular and molecular characterization of SVF and in vitro-expanded ADSC in larger SSc cohorts that include also different disease subsets might help in identifying and predicting which patients will be more likely to benefit from an autologous therapeutic approach.
